# Identification of Small-Molecule Inhibitors of the Salmonella FraB Deglycase Using a Live-Cell Assay

**DOI:** 10.1128/spectrum.04606-22

**Published:** 2023-02-21

**Authors:** Anice Sabag-Daigle, Erin F. Boulanger, Pankajavalli Thirugnanasambantham, Jamison D. Law, Alex J. Bogard, Edward J. Behrman, Venkat Gopalan, Brian M. M. Ahmer

**Affiliations:** a Department of Microbial Infection and Immunity, The Ohio State University, Columbus, Ohio, USA; b Department of Chemistry and Biochemistry, The Ohio State University, Columbus, Ohio, USA; Universidad Andres Bello

**Keywords:** FraB, *Salmonella*, antimicrobial agents, enzyme kinetics, high-throughput screen, sugar-phosphate toxicity

## Abstract

Nontyphoidal salmonellosis is one of the most significant foodborne diseases in the United States and globally. There are no vaccines available for human use to prevent this disease, and only broad-spectrum antibiotics are available to treat complicated cases of the disease. However, antibiotic resistance is on the rise and new therapeutics are needed. We previously identified the Salmonella *fraB* gene, that mutation of causes attenuation of fitness in the murine gastrointestinal tract. The FraB gene product is encoded in an operon responsible for the uptake and utilization of fructose-asparagine (F-Asn), an Amadori product found in several human foods. Mutations in *fraB* cause an accumulation of the FraB substrate, 6-phosphofructose-aspartate (6-P-F-Asp), which is toxic to Salmonella. The F-Asn catabolic pathway is found only in the nontyphoidal Salmonella serovars, a few Citrobacter and Klebsiella isolates, and a few species of Clostridium; it is not found in humans. Thus, targeting FraB with novel antimicrobials is expected to be Salmonella specific, leaving the normal microbiota largely intact and having no effect on the host. We performed high-throughput screening (HTS) to identify small-molecule inhibitors of FraB using growth-based assays comparing a wild-type Salmonella and a Δ*fra* island mutant control. We screened 224,009 compounds in duplicate. After hit triage and validation, we found three compounds that inhibit Salmonella in an *fra*-dependent manner, with 50% inhibitory concentration (IC_50_) values ranging from 89 to 150 μM. Testing these compounds with recombinant FraB and synthetic 6-P-F-Asp confirmed that they are uncompetitive inhibitors of FraB with *K_i_*′ (inhibitor constant) values ranging from 26 to 116 μM.

**IMPORTANCE** Nontyphoidal salmonellosis is a serious threat in the United States and globally. We have recently identified an enzyme, FraB, that when mutated renders Salmonella growth defective *in vitro* and unfit in mouse models of gastroenteritis. FraB is quite rare in bacteria and is not found in humans or other animals. Here, we have identified small-molecule inhibitors of FraB that inhibit the growth of Salmonella. These could provide the foundation for a therapeutic to reduce the duration and severity of Salmonella infections.

## INTRODUCTION

The top three foodborne infections causing hospitalization in the United States are Salmonella, norovirus, and Campylobacter (1). Salmonella is the most severe and the leading cause of hospitalization and death from foodborne illness in the United States (1). Diarrhea is also within the top four causes of morbidity and mortality in developing nations, especially in children, and Salmonella is a major contributor (2–4). Salmonella enterica subspecies *enterica* includes a few typhoidal serovars that cause typhoid fever and hundreds of nontyphoidal serovars that cause gastroenteritis (5).

In humans, the nontyphoidal serovars of Salmonella cause an acute gastroenteritis that is characterized by an inflammatory diarrhea and fever (6–8). There are no vaccines for human use that protect against the nontyphoidal serovars (9, 10). For serious cases involving highly susceptible patients (the very young or elderly), invasive disease, or other clinical complications, broad-spectrum antibiotics are used. However, broad-spectrum antibiotics are not recommended for uncomplicated cases of Salmonella-mediated gastroenteritis because these antibiotics disrupt the normal microbiota and, ironically, increase susceptibility to the pathogen and can increase the duration of Salmonella shedding (11–15). It is precisely these uncomplicated infections that cause great discomfort in the developed world and result in death by dehydration in developing countries. There are no drugs available to specifically decrease the duration and severity of an intestinal Salmonella infection. For the existing broad-spectrum antibiotics, multidrug resistance is prevalent and increasing (16, 17), thus necessitating innovative therapeutic approaches. In fact, both the CDC and the WHO have recently released reports stating that new drugs are needed for the nontyphoidal Salmonella serovars due to increasing fluoroquinolone resistance (18, 19). We take a first step here to address this gap. Our long-term goal is to develop narrow-spectrum antibiotics that, for the first time, could be recommended for uncomplicated cases in which the patient is seeking treatment to reduce the severity and duration of intestinal symptoms. In this study, we describe our first efforts to identify small molecules that inhibit FraB, a deglycase target that we recently identified (20–23).

**FIG 1:**
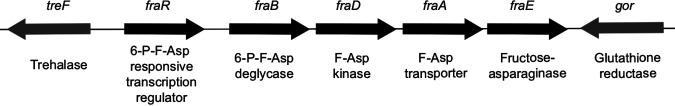
Map of the *fra* locus of Salmonella enterica. The five genes of the *fra* locus are shown as black arrows. The *gor* and *treF* genes are shown as gray arrows and are conserved throughout the *Enterobacteriaceae*, while the *fra* locus is not, suggesting that the *fra* locus was horizontally acquired.

FraB is encoded by the *fra* locus (Fig. 1). Bioinformatics analysis suggests that *fraR* is a monocistronic transcript, while *fraBDAE* constitute an operon. These five genes are not present in Escherichia coli and appear to represent a horizontal acquisition that is inserted between the *gor* and *treF* genes of the Salmonella chromosome. These genes confer the ability to use fructose-asparagine (F-Asn) as the sole carbon and nitrogen source (20). F-Asn, an Amadori compound, is found in fruits and vegetables, especially if they have undergone a heat drying process (24). We have confirmed that only a few other bacteria, primarily from the class *Clostridia*, encode Fra homologs and can indeed utilize F-Asn (25). Upon uptake, F-Asn is first converted by the FraE asparaginase to F-Asp, which is then phosphorylated by the FraD kinase to form 6-phosphofructose-asparate (6-P-F-Asp) (26). In the final step, the FraB deglycase converts 6-P-F-Asp to aspartate and glucose-6-P (22).

**FIG 2:**
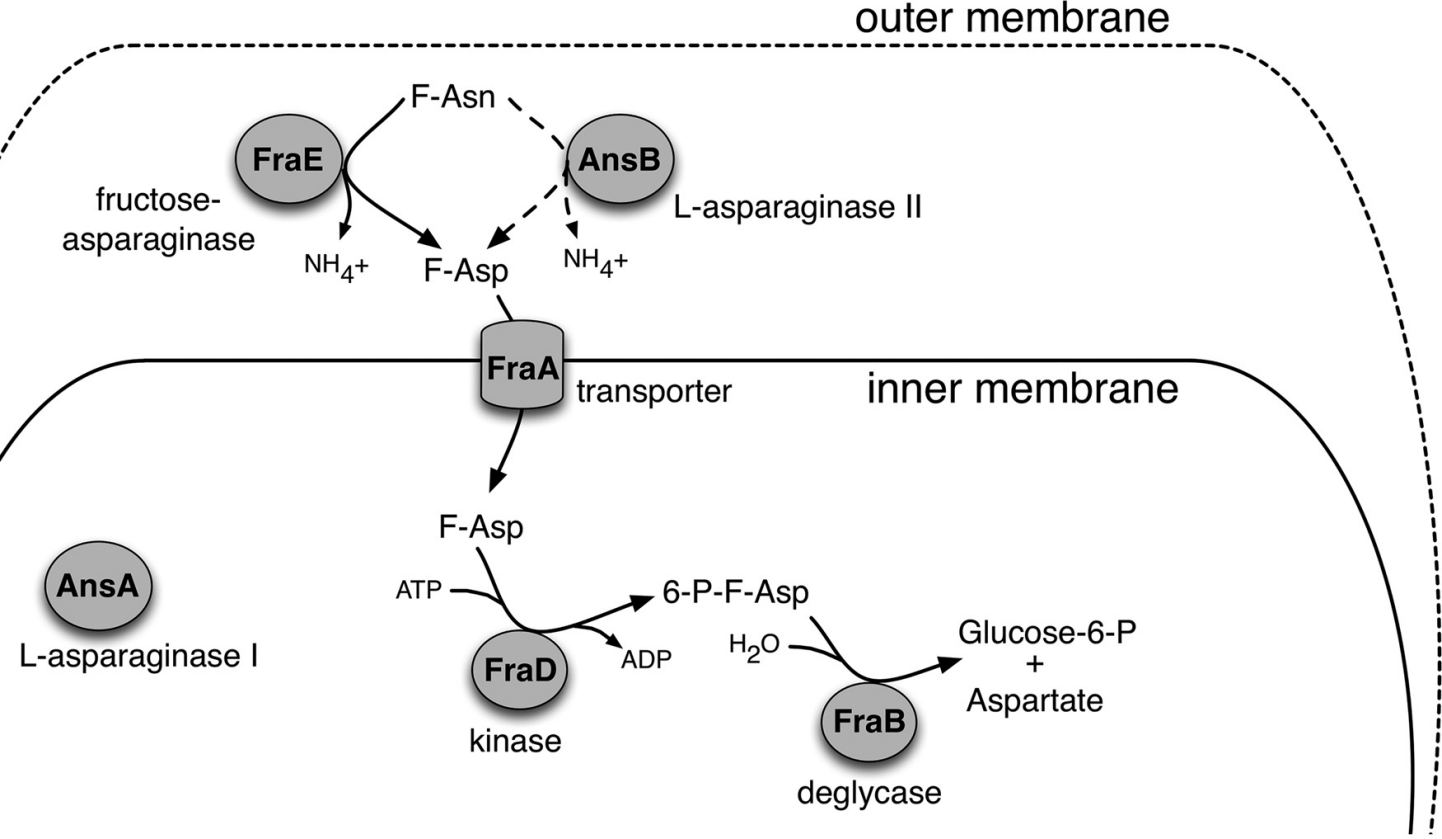
Fra protein localization and function. A proteomic survey of subcellular fractions of Salmonella previously identified FraB (the deglycase) as cytoplasmic and FraE (the asparaginase) as periplasmic (42). See the text for details of individual transformations in this catabolic pathway. This figure is adapted from reference 43.

A *fraB*::Kan mutant of Salmonella is dramatically attenuated in several mouse models (20). This phenotype is not due to the role of F-Asn as a nutrient but is rather due to the toxic accumulation of 6-P-F-Asp, a metabolite in the F-Asn utilization pathway (Fig. 2) (21, 23). Several lines of evidence support this claim. First, a mutant lacking *fraD*, or the entire *fra* island, can grow in minimal medium containing glucose and F-Asn, while a *fraB*::Kan mutant cannot (21). Second, mass spectrometry studies established that 6-P-F-Asp accumulates to high levels in the *fraB*::Kan mutant but not in a *fra* island deletion mutant (21). Third, a *fraB*::Kan mutant is severely attenuated in streptomycin-treated Swiss Webster mice, while a *fraD* mutant and a mutant lacking the entire *fra* island are not (21). Since none of these mutants can grow on F-Asn, this result demonstrates that a failure to utilize F-Asn in this particular mouse model is not responsible for the attenuation. We hypothesize that strong inhibitors of FraB activity would have the same effect as mutation of *fraB* and would be effective in inhibiting Salmonella during infection. Therefore, we performed a high-throughput screen (HTS) to identify small-molecule inhibitors of Salmonella FraB, and we report our findings here.

## RESULTS

A Salmonella *fraB* mutant fails to grow in the presence of F-Asn (20, 21). FraB catalyzes the conversion of 6-P-F-Asp to glucose-6-P and aspartate (22). In the absence of *fraB*, 6-P-F-Asp accumulates and inhibits growth through an unknown mechanism (21). We refer to this inhibition of growth as intoxication. These observations led to the hypothesis that a small-molecule inhibitor of FraB enzyme activity would intoxicate Salmonella in the presence of F-Asn. To test this hypothesis, we performed a high-throughput screen of small molecules at the ICCB-Longwood facility at Harvard Medical School to identify inhibitors of FraB. We utilized a live-cell assay with Salmonella growth as the readout for FraB inhibition.

**FIG 3:**
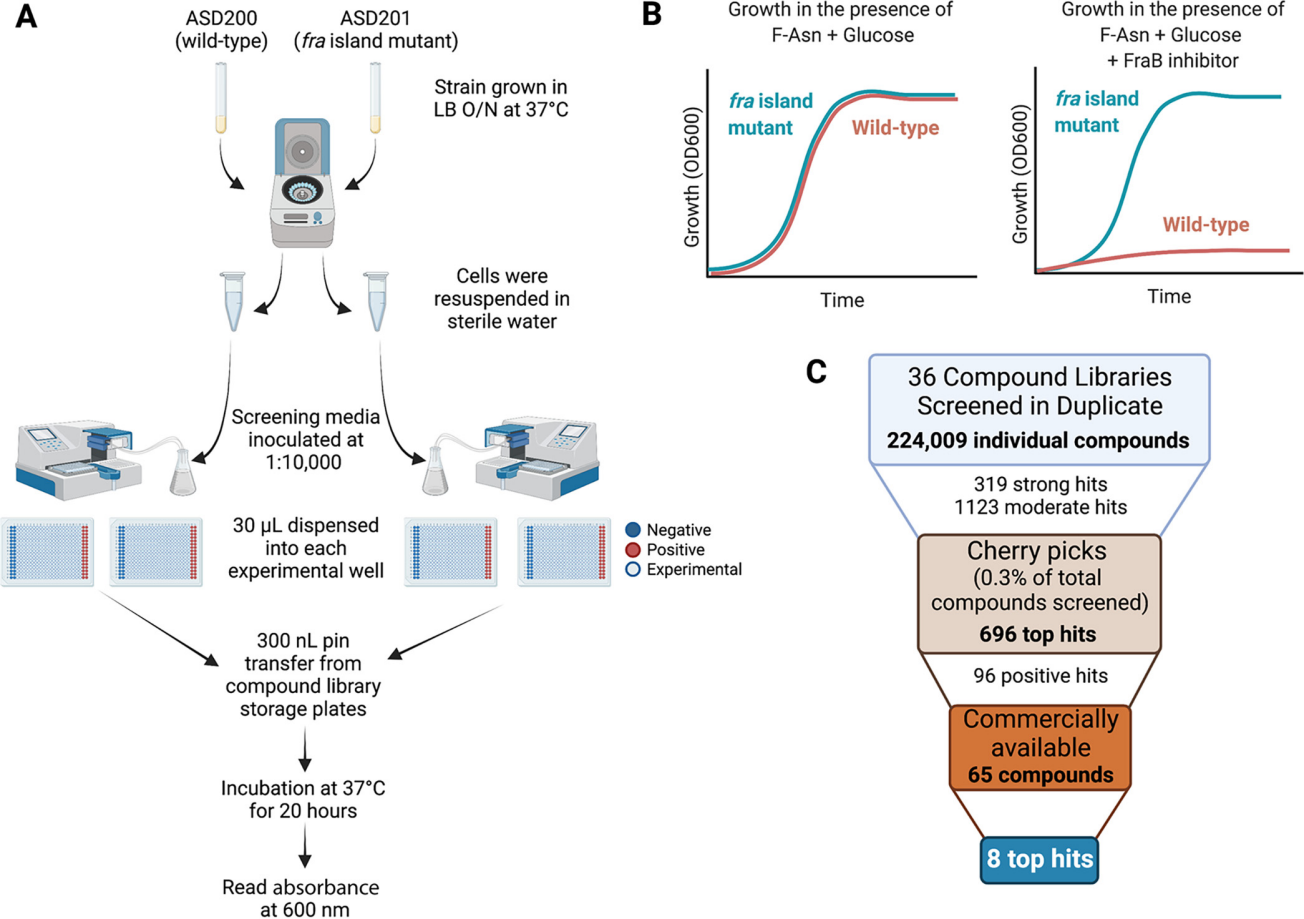
(A) Schematic of the HTS used in this study. O/N, overnight. (B) Expected growth curves of the wild-type Salmonella, ASD200, and the isogenic *fra* island mutant, ASD201, in M9 minimal medium containing glucose and F-Asn with or without a potential FraB inhibitor. (C) Flow chart of the number of compounds tested at each step.

**FIG 4:**
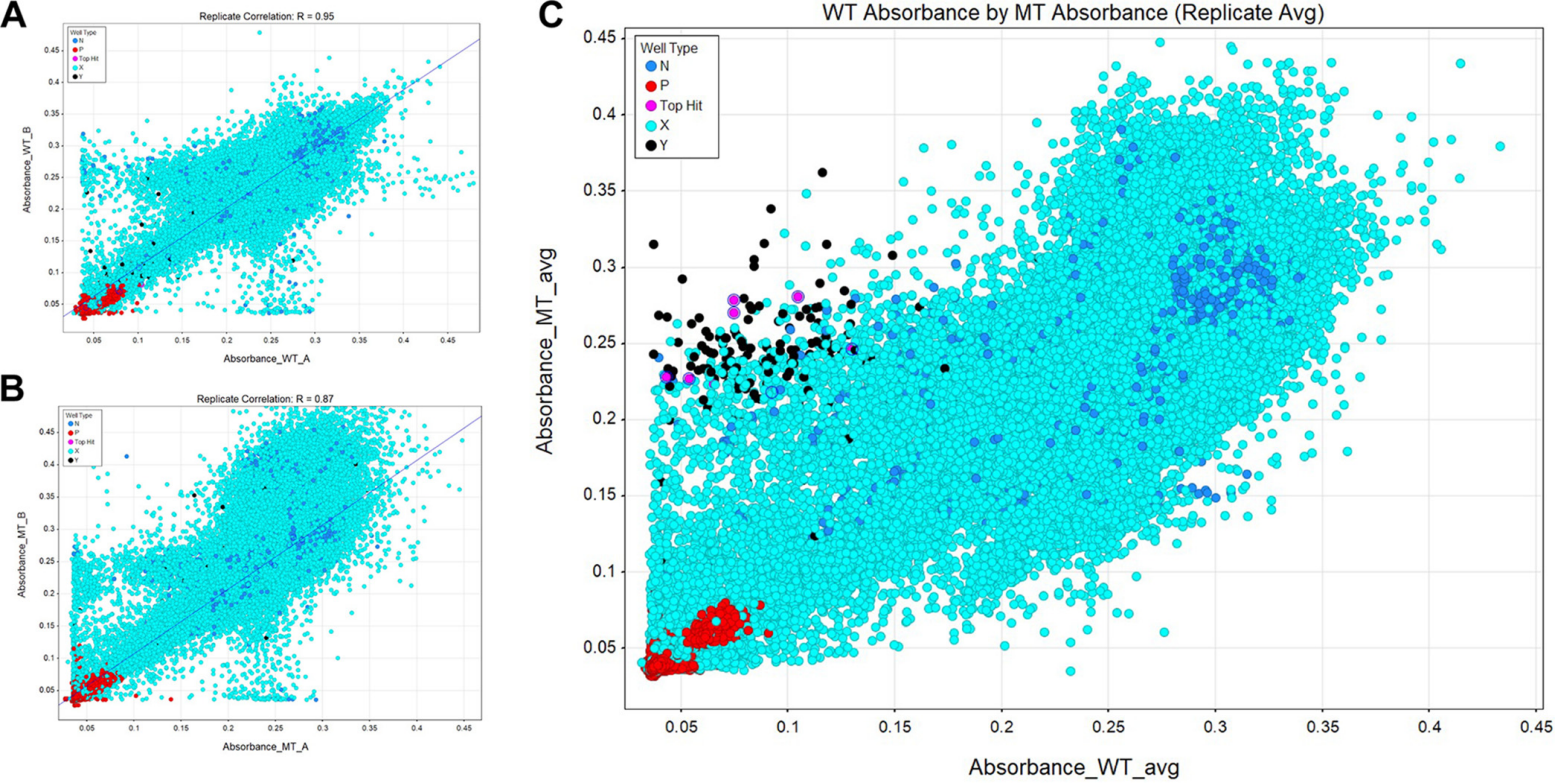
Correlation plot of two replicates of the *A*_600_ values of the wild-type strain ASD200 (A), the *fra* island mutant strain ASD201 (B), and all experimental wells for both strains screened (C; *x* axis, wild-type and *y* axis, mutant). In panel C, candidate hits are identified by a low absorbance for the wild-type and a high absorbance for the mutant. Color key to filled circles: the experimental wells are shown in light blue, cherry-picked compounds are in black, and the 8 top hits are in pink. Negative- and positive-control wells are indicated in blue and red, respectively.

It is likely that most inhibitors of growth are cytotoxic compounds that act independently of the *fra* locus. To identify *fra*-dependent inhibitors of growth, we therefore screened in parallel a second strain that lacks the entire *fra* locus (*fraR fraBDAE*), which we refer to as the *fra* island mutant. The deletion of this genomic island prevents the formation of 6-P-F-Asp, and therefore, this strain should be unaffected by inhibitors of FraB. The screening was conducted in M9 minimal medium containing 5 mM glucose and 1 mM F-Asn. Glucose provides the carbon and energy source, while F-Asn is required for growth inhibition if a FraB inhibitor is present (21). Cells were inoculated into the growth medium in 384-well plates, and then compounds were pinned into the wells at a typical concentration of 150 μM. We recognize that quantitative HTS, which entails concentration-response curves, enhances the accuracy of hit calling (27). However, substrate (F-Asn) limitations and cost considerations prohibited such a titration-based approach in our HTS. The plates were incubated at 37°C overnight (20 h), at which point the growth was measured using *A*_600_ (Fig. 3). We screened 224,009 compounds in duplicate. Duplicate replicates of the wild-type and mutant strains exhibited high correlations of *R* = 0.95 and *R* = 0.87, respectively (Fig. 4).

**FIG 5:**
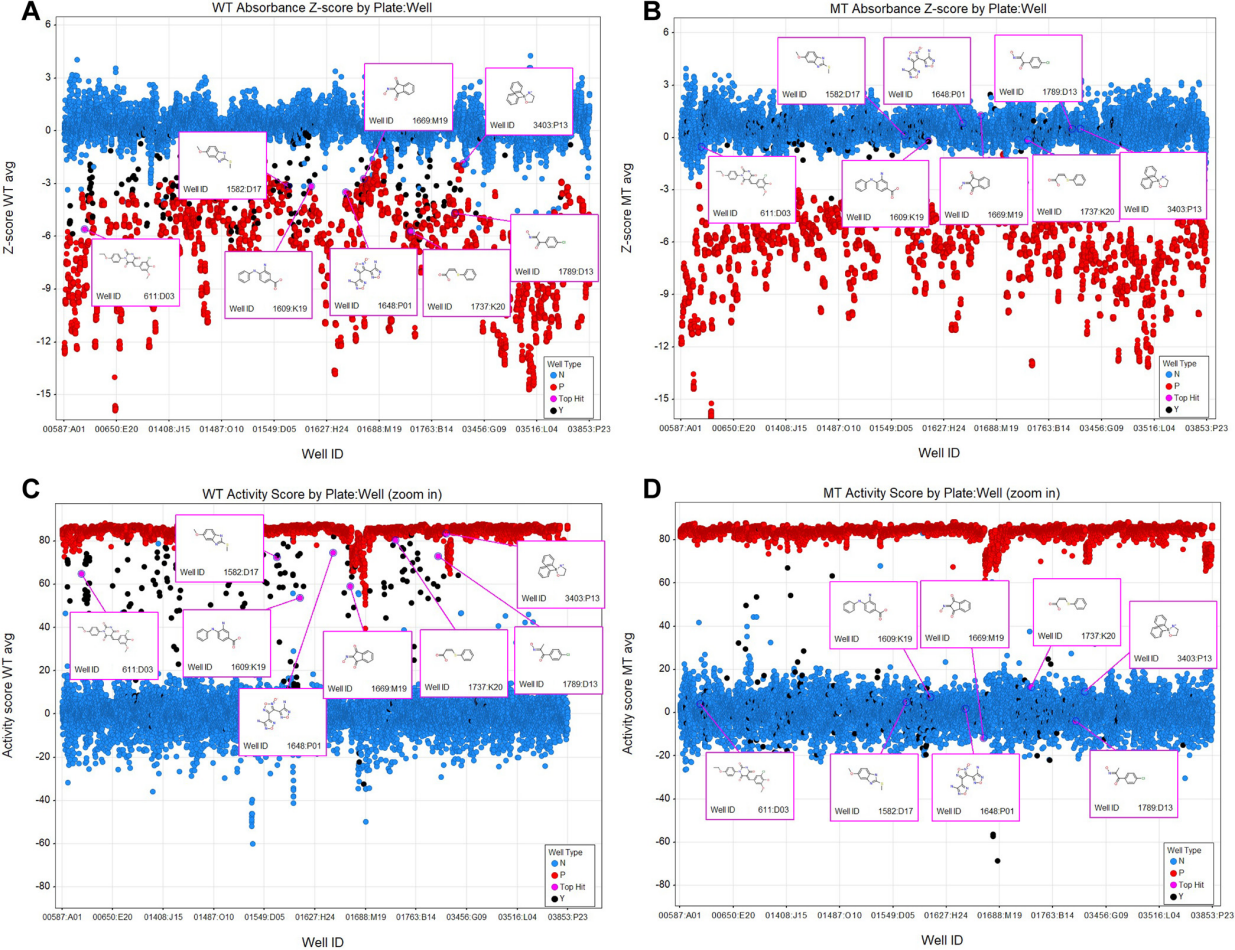
(A, B) Z-score of each cherry-picked compound on the wild-type ASD200 (A) and on the *fra* island mutant ASD201 (B). (C, D) Activity score of each cherry-picked compound on the wild-type ASD200 (C) and on the mutant ASD201 (D). Color key to filled circles: the cherry-picked compounds are depicted in black, and the 8 top hits are in pink. Negative- and positive-control wells are indicated in blue and red, respectively. All other experimental wells were removed for clarity.

**FIG 6:**
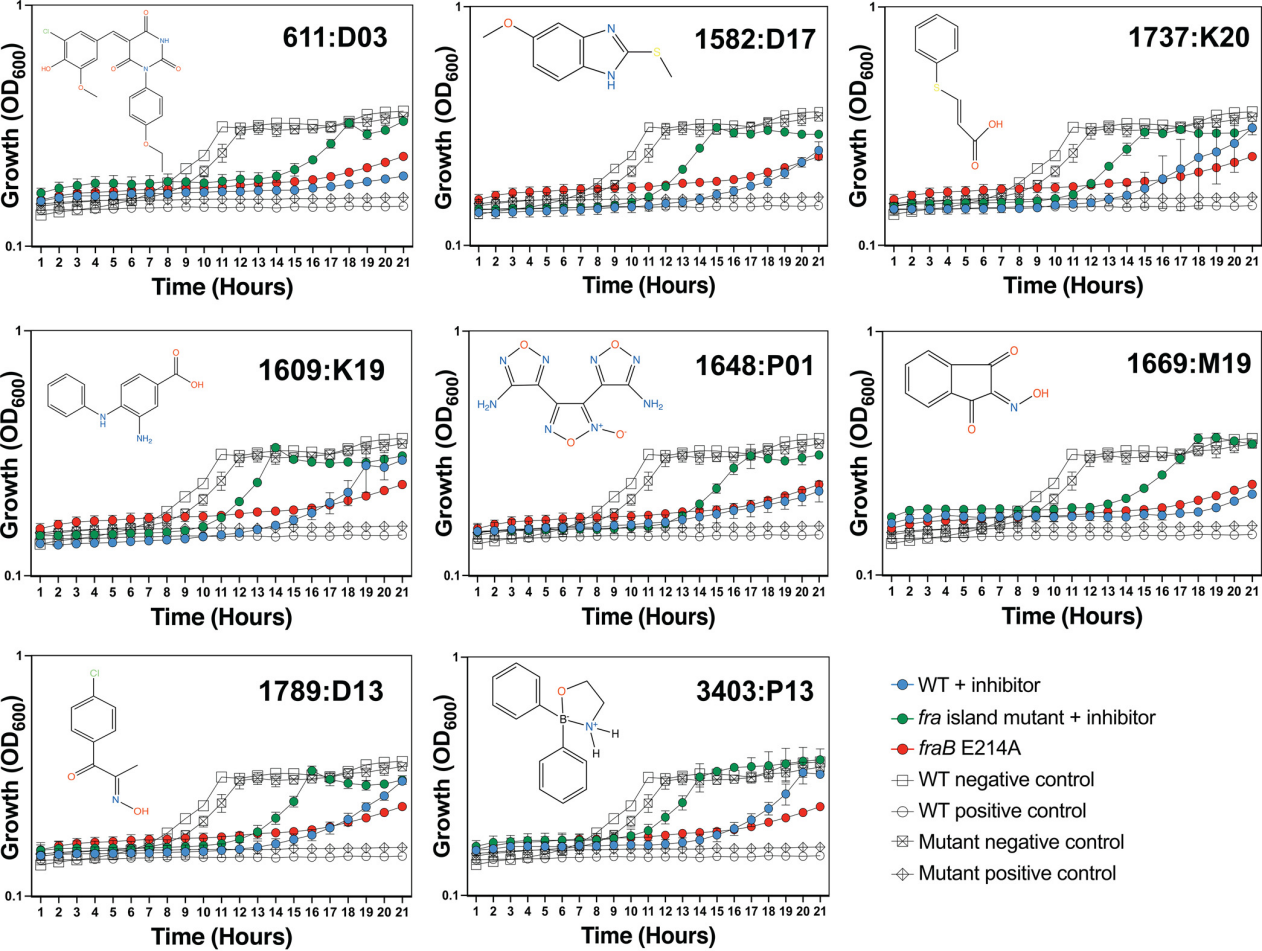
Growth curves in the presence of different inhibitors. The structure of each inhibitor is shown along with the corresponding growth curve of wild-type (ASD200, blue line), *fra* island mutant (ASD201, green line), and *fraB* E214A mutant (ASD1312, red line) strains cultured in the presence of 250 μM compound depicted (except for 3403:P13 at 125 μM). The positive control is growth in chloramphenicol, and the negative control is growth in 1.9% (vol/vol) DMSO. Growth was measured by monitoring *A*_600_ in a Molecular Devices SpectraMax i3x. Symbols and error bars in the growth curves represent the mean values and standard deviations calculated from three technical replicates associated with a single biological trial.

Of the compounds screened, 319 were considered strong hits and 1,123 were moderate hits (see Materials and Methods for scoring). The top 696 hits (0.3% of the tested compounds) were cherry picked and shipped to us at OSU for further testing. Of these, 96 were confirmed as hits at OSU. From this pool, 69 were purchased commercially and three found to be insoluble, leaving 66 that were tested again in the live-cell assay. While the initial screening at the ICCB-Longwood facility and the screening of the cherry picks at OSU were performed by measuring growth at a final 20-h time point, the effects of the purchased compounds were measured hourly, given the smaller number of compounds. After screening the 66 purchased compounds with the live-cell assay, 8 were considered hits (Fig. 5). Interestingly, the compounds only hit at select points in the growth curve (Fig. 6; see Discussion). A mutant of Salmonella with a *fraB* E214A mutation (a change of E to A at position 214) in the chromosome (ASD1312) was included in these experiments. This mutant strain harbored a catalytically inactive FraB variant and served as a proxy for how a cell with a fully inhibited FraB enzyme might grow (22). The growth of the wild-type Salmonella in the presence of all eight inhibitors was very similar to the growth of the Salmonella *fraB* E214A mutant (Fig. 6), suggesting that the FraB enzyme is inhibited within the wild-type cells. It is noteworthy that these eight inhibitors identified in our screen have not been identified as *bona fide* actives in any previous screen.

All eight inhibitors appeared to have both a *fra*-dependent and a *fra*-independent component to their activity (Fig. 6). The *fra*-dependent activity was evident from our finding that the *fra*-positive strain was inhibited more than the *fra* island mutant. The *fra*-independent activity was apparent from our observation that the *fra* island mutant did not grow as well in the presence of inhibitor as the dimethyl sulfoxide (DMSO) control. The 50% inhibitory concentration (IC_50_) and IC_90_ values of each of the eight compounds on live cells are shown in Table 1, with the IC_50_s for all of them ranging between ~90 and 180 μM.

**TABLE 1:** Characteristics of the top 8 inhibitors from the live-cell assay

PubChem compound ID^*a*^	Structure image	Source ID^*b*^	Library	Vendor	Vendor reagent ID	Value (95% CI) (μM) with live cells^*c*^		Mean value ± SE (μM) for^*i*^:	
						IC_50_	IC_90_	IC_50_ with recombinant FraB^*d*^	*K_i_*′ with FraB; mode of inhibition^*e*^
5442752	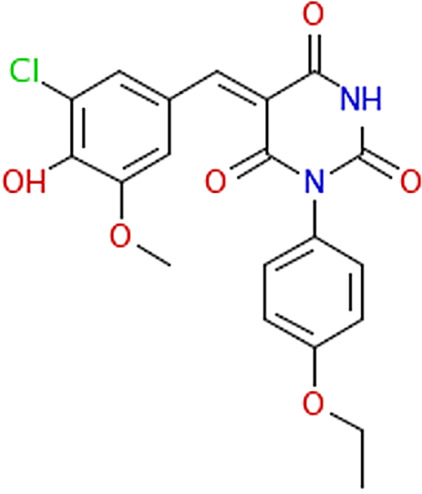	611:D03^*f*^	ChemDiv1	ChemDiv	4058-0407	100 (79–130)	240 (158–443)	ND^*g*^	ND
838366	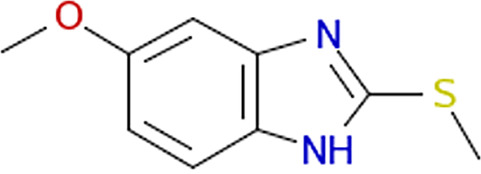	1582:D17	ChemBridge3	ChemBridge	5540846	143 (124–167)	318 (236–446)	NI^*h*^	ND
773821	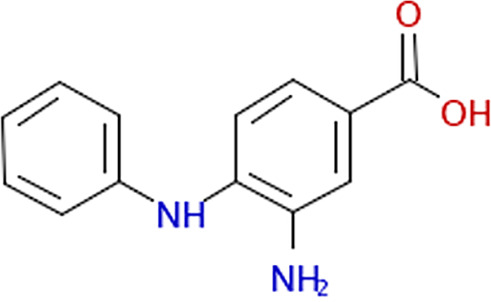	1609:K19	ChemDiv4	ChemDiv	2057-0008	155 (130–187)	411 (287–619)	114 ± 3.8	116 ± 27; uncompetitive
394430	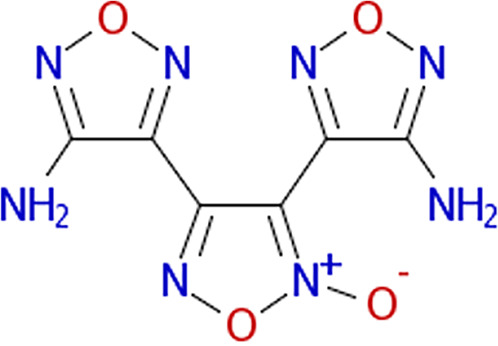	1648:P01	ChemDiv4	ChemDiv	R052-2211	89 (70–118)	259 (153-548)	>500	ND
316190	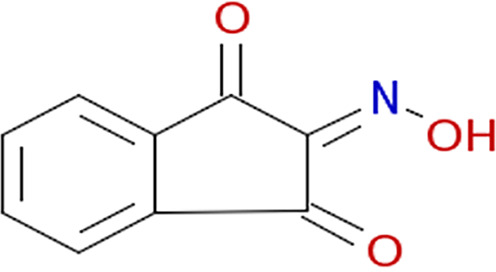	1669:M19	Maybridge5	Maybridge	SPB 00184	109 (73–171)	297 (159–1,100)	133 ± 0.8	26 ± 4; uncompetitive
1547667	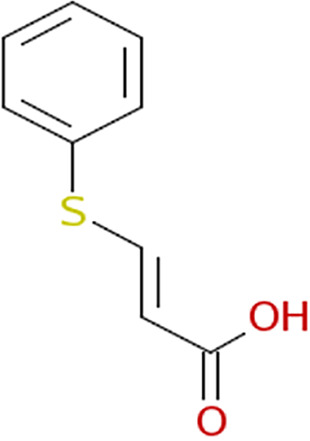	1737:K20	Enamine2	Enamine	T5429666	121 (73–208)	503 (213–1,500)	NI	ND
9603109	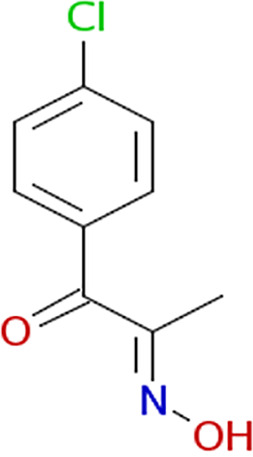	1789:D13	Enamine2	Enamine	T5503425	183 (160–207)	348 (278–456)	NI	ND
13317552	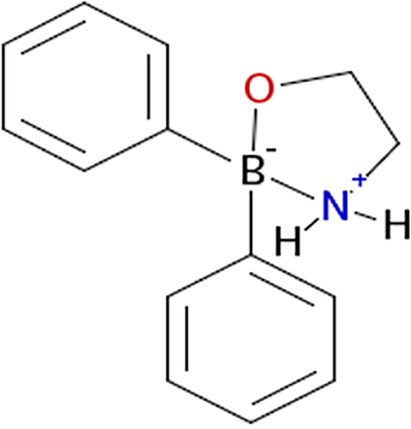	3403:P13	BiomolICCBL–2012	Enzo Life Sciences	AC-1557	89 (73–108)	184 (130–284)	35 ± 0.9	27 ± 6 uncompetitive

a ID, identification number.

b ICCB-Longwood designation, including the source plate ID and the well ID.

c Concentration that inhibits growth to 50% or 90%. CI, confidence interval.

d Concentration that inhibits FraB activity by 50%. The mean and standard error were determined using data from two independent experiments.

e Concentration of the inhibitor required for dissociation of the enzyme-substrate-inhibitor complex. The mean and standard deviation were determined from three or more independent trials.

f A PAINS compound.

g ND, not determined.

h NI, no inhibition observed with 1 mM of the specified compound.

i IC_50_ uses SE and *K_i_*′ uses SD.

**FIG 7:**
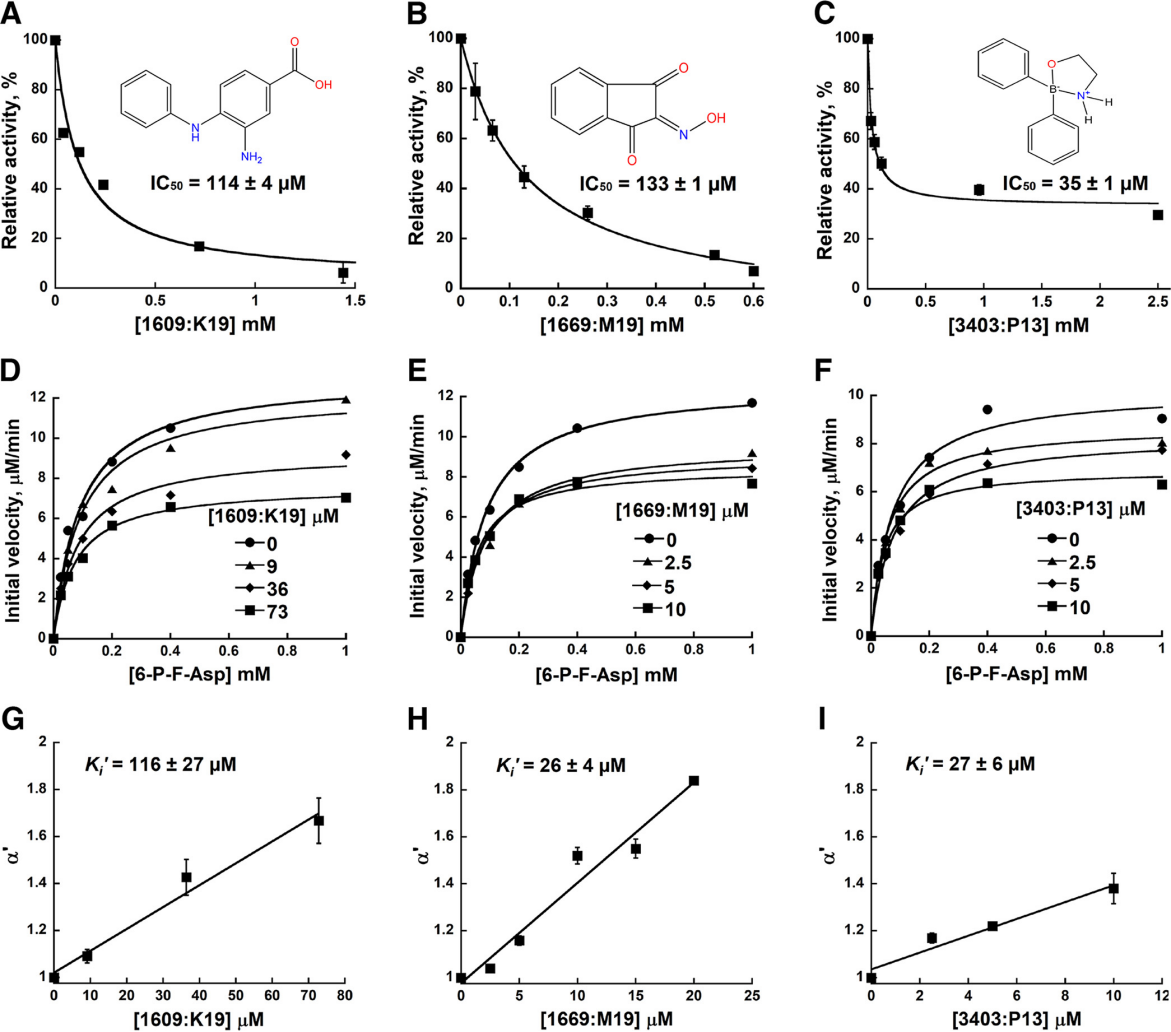
Characterization of the inhibition of Salmonella FraB by 1609:K19 [3-amino-4-(phenylamino)benzoic acid], 1669:M19 (triketoindan-2-oxime), and 3403:P13 (2,2-diphenyl-1,3,2-oxaazaborolidine internal salt). (A to C) Determination of the IC_50_ values for each inhibitor. (D to F) Michaelis-Menten analyses with three different concentrations for each inhibitor. A representative plot from one of the three or more independent measurements is depicted here. The curve fit errors for *K_m_* and *k*_cat_ did not exceed 23% and 10%, respectively, in any trial. All curve fits had correlation coefficients of ≥0.98. (G to I) The *k*_cat_ values were used to determine the α′ values, which in turn were used in the equation α′ = 1+ [*I*]/*K_i_*′ to obtain the respective *K_i_*′ values. The error bars reflect the standard deviations of the mean α′ values determined from three or more Michaelis-Menten analyses similar to the representative data shown in panels D to F.

These eight compounds were also tested in an *in vitro* assay with purified FraB enzyme and synthetic 6-P-F-Asp as the substrate (Fig. 7). For determining the IC_50_ value of each inhibitor, we used a concentration-response plot. The relative activity of FraB was plotted versus different concentrations of the inhibitor. At least two independent trials were conducted for each compound. Our results indicate that three of the eight compounds were inhibitory to FraB, with IC_50_ values between ~35 and 130 μM. Three of the compounds did not inhibit even at 1,000 μM, one was likely to have an IC_50_ of >500 μM, and one was a pan-assay interference compound (PAINS) (28); therefore, these five compounds were not considered further (Table 1).

For determining the *K_i_* values and the type of inhibition, we performed Michaelis-Menten analyses with six different concentrations of 6-P-F-Asp in the absence of inhibitor or in the presence of three different concentrations of the test compound. The initial velocities were calculated as described above, and the kinetic parameters (*K_m_* and *k*_cat_) were obtained by fitting the data using KaleidaGraph (Synergy Software). The individual *K_m_* and *k*_cat_ values yielded α′ values, which in turn were used in the equation α′ = 1+ [*I*]/*K_i_*′, where *I* is the inhibitor and *K_i_*′ is the inhibitor constant, to obtain the respective *K_i_*′ value. The *K_i_*′ values ranged between 26 and 116 μM for the three compounds (Fig. 7), with all three behaving as uncompetitive inhibitors (Fig. 7).

## DISCUSSION

From our cell-based HTS, we have successfully identified three compounds that inhibit wild-type Salmonella in an *fra*-dependent manner and inhibit recombinant FraB *in vitro*. These compounds also have a slight inhibitory effect on cells lacking the *fra* island, suggesting off-target (albeit minimal) effects. All three hits are uncompetitive inhibitors, indicating that they do not inhibit in the absence of substrate and likely bind outside the active site. Structural studies are required to gain insights into the binding pockets that accommodate these inhibitors. Although the *K_i_*′ (enzyme-based) and IC_50_ (cell-based) values are in the micromolar range for our winners (Table 1), specificity is as important a consideration as potency while choosing hits for follow-up studies (29). We are therefore buoyed by our findings that the mechanism of action of our top hits entails the intended target (FraB).

Some other findings merit elaboration. Despite robust replicates and stringent scoring, we found that hit triage and validation whittled the list of cherry picks from the HTS by 7-fold (from 696 to 96). We consider a few reasons for this finding. Foremost, it is difficult to know if the compounds tested in the HTS were indeed assayed at the specified molarity, given the potential for solvent evaporation or precipitation from repeated freeze-thaw cycles of the master stock plates. This contention is also supported by an unexpected finding that the final list of 8 winners score as hits only at select points in the growth curve. In fact, the two strains often achieved the same absorbance at the 20-h time point when tested in-house (Fig. 6). Since these same compounds scored as hits in the initial large-scale HTS, which sought to distinguish hits and non-hits using the 20-h time point, it is conceivable that molarity differences may account for the discord that we observed. To ensure that *bona fide* hits are not missed, it would have been useful to take measurements at two different time points, a modification that we will consider in the future. Second, the large-scale HTS entailed the use of 384-pin transfer pins (300 nL/well), while the cherry-picked validations were performed in-house with multichannel pipettes (3 μL/well), identifying a potential variable pertaining to wettability and transfer. Finally, the HTS at the Longwood facility used compound stocks prepared in 100% (vol/vol) DMSO, while the cherry-picked samples were in 19% (vol/vol) DMSO. Self-association of DMSO (at 100%) may have affected the effective concentrations of the compounds.

As a next step to this investigation, we plan to optimize the hit compounds described in this report through synthesis of derivatives. Optimization and characterization of these small-molecule inhibitors of FraB may pave the way to a drug that could potentially decrease the duration and severity of an acute Salmonella infection while leaving the normal microbiota intact. Because no animals, and very few bacteria, have a similar FraB-like target, we expect these FraB inhibitors to have little to no effect on the human (or other vertebrate) host and minimal effect on the intestinal microbiota. While such an advance could save lives in the developing world, it would also be greatly appreciated by patients suffering a 7-day Salmonella infection in the developed world. Of course, we recognize that an initial diagnosis of the causative agent of the diarrhea prior to treatment with species-specific drugs may be unlikely. Instead, for future generations, a viable clinical strategy might include prescribing a cocktail of species-specific drugs, targeting a variety of bacteria and viruses, to treat cases of human diarrhea while minimizing disruption of the healthy microbiota. Ideally, there would be several drugs targeting each potential microbe, and probiotics and prebiotics would be included to enhance the treatment efficacy and recovery. The scientific community should be able to accomplish this goal in the near future, and a drug targeting Salmonella FraB could be one component of this cocktail. Furthermore, we and others have identified numerous other metabolic intermediates that are toxic to bacterial/fungal cells and could be induced using similar methods (23, 30).

## MATERIALS AND METHODS

### Strains and media.

All strains used in this study (Table 2) are derivatives of Salmonella enterica serovar Typhimurium strain ATCC 14028 (hereafter referred to as 14028). All Salmonella strains were routinely grown in LB broth, Miller (Fisher BioReagents). High-throughput screening (HTS) was conducted in M9 minimal medium containing 5 mM glucose and 1 mM fructose-asparagine (31, 32).

**TABLE 2:** Bacterial strains used in this study

Strain	Genotype	Reference or source
ATCC 14028	Wild-type Salmonella enterica serovar Typhimurium	American Type Culture Collection
ASD200	ATCC 14028 ΔSPI1 ΔSPI2	32
ASD201	ATCC 14028 ΔSPI1 ΔSPI2 Δ(*fraR fraBDAE*)*4*::Kan	32
ASD1312	ATCC 14028 *fraB* E214A	22

### Synthesis of fructose-asparagine.

Since F-Asn is not commercially available at a reasonable cost, we synthesized it using a procedure that we previously described (33). However, attempts to scale up our synthesis of fructose-asparagine to the gram scale were not successful at the column-separation stage. We report here a method that eliminates the column step and results in a final yield of approximately 50%.

d-Glucose (175 g, 0.97 mol), l-asparagine monohydrate (32 g, 0.21 mol), malonic acid (9 g, 0.086 mol), and sodium bisulfite (9 g, 0.086 mol) were added to 250 mL of water at 90°C with stirring. A small amount of undissolved material was removed by filtration, and then 250 mL ethylene glycol was added. About 90% of the water was then removed by rotary evaporation at 50°C for 90 min. The homogeneous mixture was incubated at 65°C in an unstoppered flask for 12 to 15 h. For best yield, the color should be dark yellow to light amber, but not darker (*A*_350_ of ~8). Water (25 mL) was then added to decrease the viscosity of this solution before Dowex 50 ×8 H^+^, 200 to 400 mesh (240 g) chromatography. Based on 1.7 milliequivalents/mL of the damp resin, this Dowex amount (240 g) represents a 1.5-fold excess of the calculated equivalents of the product. Stirring was continued for 15 min. The slurry was then filtered on a Bϋchner funnel and washed thoroughly with water. The product and excess asparagine were eluted by using ~30 mL of 7 M ammonium hydroxide, which was added slowly until the solution turned basic. Filtration and washing with a small amount of water yielded the effluent containing the product, excess asparagine, and ammonia. The ammonia was removed by rotary evaporation at 22°C. Drying was completed by rotary evaporation at 50°C followed by azeotrope treatment with ethanol. Finally, the product was extracted with acetonitrile in a Soxhlet apparatus for ~2 h to remove any residual ethylene glycol (detected by nuclear magnetic resonance [NMR], ca. 5.6 ppm) and then dried again.

The separation of F-Asn and asparagine is based on their differential solubilities in water and methanol. The solubility of asparagine in the two solvents has been documented (34–36). F-Asn is more soluble in both. When 0.6 g of the crude material was dissolved in 2 mL of water and 2 mL methanol was added dropwise with stirring, a precipitate formed. Upon cooling overnight and centrifugation, a clear supernatant was obtained. This supernatant was analyzed for residual asparagine using thin-layer chromatography (TLC) on silica with a one-phase *n*-butanol, acetic acid, water system (37). Additional water-methanol separations were used, as necessary. The proton NMR spectrum should show a 6:2:2 ratio for the three groups of resonances due to (i) the 3-, 4-, 5-, and 6-sugar protons plus the alpha proton of the asparagine residue, (ii) the two 1′-deoxy protons of the sugar, and (iii) the two beta protons of the asparagine residue, respectively. In addition to the spectrum being complicated by the four ring forms (33), the 1′-deoxy protons of the sugar exchange during the NMR experiment with the D_2_O solvent. Thus, it is better to rely on the ratio of the other two groups of resonances (specified as i and iii above). Using these new preparations of F-Asn, we obtained IC_50_ values of ~30 μM on *fraB* mutants of Salmonella, similar to the ~20 μM that we previously reported using F-Asn made with the small-scale synthesis route (33). We also note that the synthesis of Amadori compounds has been described in two recent publications, which closely examine the role of water and advance our previous knowledge (38, 39).

### HTS.

We performed HTS on two separate occasions at the ICCB-Longwood facility. The protocols differed slightly, but the vast majority of compounds were screened during the second visit and all of the final hits came from the second HTS effort. Thus, only the protocols for the second screen are described here. Also, for safety reasons, the strains we use for screening lacked Salmonella pathogenicity islands 1 and 2 (SPI1 and SPI2) (32), neither of which has any effect on F-Asn intoxication.

ASD200 (14028 ΔSPI1 ΔSPI2) and ASD201 [14028 ΔSPI1 ΔSPI2 Δ(*fraR fraBDAE*)] were grown overnight in LB broth at 37°C with continuous shaking. Overnight cultures were centrifuged at 14,000 × *g* for 1 min and resuspended in an equal volume of sterile water. This washed overnight culture was further diluted 1:100 in sterile water. Each strain was then added to separate flasks of screening medium at a 1:100 dilution for a final dilution of 1:10,000. Each individual strain in screening medium was then dispensed at 30 μL per well into 384-well plates (Corning 3701) using a multidrop combi dispenser equipped with a standard tube dispensing manifold (Thermo Scientific). For all screening plates, positive controls consisted of either ASD200 or ASD201 in screening medium containing chloramphenicol dissolved in DMSO at a final chloramphenicol concentration of 50 μg/mL and a final DMSO concentration of 1% (vol/vol).

The negative controls consisted of ASD200 or ASD201, which were grown in screening medium containing a final concentration of 1% DMSO (vol/vol). Concentrations of DMSO of less than 5% (vol/vol) did not affect the growth of either strain (data not shown). Two columns (C1 and C2) of positive control wells and two columns (C23 and C24) of negative control wells were included in each 384-well plate; aliquoting entailed the use of an 8-channel pipette. Subsequently, 300 nL of a test compound was pinned into each well using an Epson compound transfer robot (SGM612). Each compound was pinned into four plates total, two containing ASD200 and two containing ASD201. Nearly all compounds were at a stock concentration of 5 mg/mL, resulting in an assay concentration of 50 μg/mL (the molar concentrations ranged from 10 μM to 460 μM but were typically ~150 μM). Lids were placed on all plates before the plates were incubated at 37°C for 20 h. After incubation, the lids were removed from all plates, the plates were placed in a plate stacker, and absorbance at 600 nm was measured using a Perkin Elmer EnVision plate reader. The total list of libraries screened is in file 1 in the supplemental material. Because not all plates from the listed libraries were screened, the total number of compounds screened is not equal to the total number of compounds available in the libraries listed. Results from pilot screens with representative plates from each library shaped our decision on the total number of plates chosen from each library for follow-up screening.

### Data analysis.

Each plate was scored separately. Every compound was assayed in duplicate with strain ASD200 (14028 ΔSPI1 ΔSPI2) and in duplicate with strain ASD201 [14028 ΔSPI1 ΔSPI2 Δ(*fraR fraBDAE*)]. To be considered a *fra*-dependent hit, the compound needed to impair the growth of ASD200 but not ASD201. A Z-score and an activity score were calculated for the two bacterial strains grown in the presence of each compound. The Z-score was calculated as the experimental value (absorbance at 600 nm) minus the average of all experimental values for that strain on the plate divided by the standard deviation of all experimental values for that strain on the plate (40). For a test compound to be considered a hit based on the Z-score, the difference between the Z-scores for ASD200 and ASD201 had to be greater than −3 to be a strong hit, greater than −2 to be a medium hit, and greater than −1 to be a weak hit. The activity score was calculated as 100 × [1 − (the experimental value/the average value obtained with all negative control wells on the plate)]. For a test compound to be considered a hit based on the activity measurement, the activity score for ASD201 must be <10 and the activity score for ASD200 must be >50 (strong hit), >40 (medium hit), or >20 (weak hit). Since each compound had four scores (duplicate Z-scores and duplicate activity scores), the four scores were added together to get a final score. This scoring was performed by assigning values of 1.0, 0.6, and 0.3 to strong, medium, and weak scores, respectively, and then adding the four together. After compiling all four together, we used the following metric: strong >3, 3> moderate > 1.6, and 1.5 > weak > 0.3.

### Screening of cherry-picked compounds.

The ICCB-Longwood facility shipped to us at OSU a total of 696 cherry picks, each of which was diluted 1:10 from neat DMSO master stocks in 10% (vol/vol) DMSO to yield a final concentration of 500 μg/mL in 19% (vol/vol) DMSO. These compounds were assayed in 384-well plates (Corning 3701) as described above, except that the total volume of medium added to the well was 27 μL and then 3 μL of diluted compound was added to each well, for a final concentration of 1.9% (vol/vol) DMSO. Each strain was inoculated at a 1:10,000 dilution. As with the original screening at ICCB-Longwood, the stock concentrations were 5 mg/mL, resulting in an assay concentration of 50 μg/mL (molar concentrations ranged from 10 μM to 460 μM, typically ~150 μM). Lids were placed on all 384-well plates, and the plates incubated at 37°C for 20 h. After incubation, the lids were removed from all plates, and *A*_600_ was measured using a Molecular Devices SpectraMax i3x. All cherry-picked compounds are listed in file 2 in the supplemental material.

### Screening of purchased compounds.

Compounds that were commercially available were ordered in 5-mg aliquots in glass vials. Compounds were resuspended to 100 mM in 100% (vol/vol) DMSO. For a working solution of 1.5 mM in 10% (vol/vol) DMSO, 5 μL of the stock [100 mM in 100% (vol/vol) DMSO] was added to 328.3 μL of 10% (vol/vol) DMSO. Purchased compounds were initially assayed at a final concentration of 150 μM and 1% (vol/vol) DMSO by adding 3 μL to 27 μL of screening medium. Each strain was inoculated at a 1:10,000 dilution. All 384-well plates were incubated at 37°C for 20 h in a Molecular Devices SpectraMax i3x, with the wells covered with Breathe-Easy film (Diversified Biotech) to prevent evaporation. During incubation, the absorbance at 600 nm was measured hourly. The purchased compounds were scored at multiple time points.

The top 8 compounds were assayed again in 384-well format, using the same procedures but with varying concentrations of compound (31.2 μM to 10 mM) to calculate the 50% and 90% inhibitory concentrations (IC_50_ and IC_90_) using Prism 9 (GraphPad) software. The 17-h time point was used for these calculations.

### FraB deglycase assays.

The substrate for FraB, 6-phosphofructose-aspartate (6-P-F-Asp) was synthesized as described previously (33). His_6_-FraB (cloned into the pET-33b vector) was overexpressed in E. coli SixPack cells (41) and purified to homogeneity as described previously (22). FraB activity in either DMSO or DMSO with dissolved compounds was assayed by using a coupled assay that affords continuous measurement of NADH produced by glucose-6-phosphate dehydrogenase (G6PD) (product number LS003997; Worthington Biochemical Corporation) (22). For the IC_50_ measurements, the assays contained 0.2 μM recombinant His_6_-FraB, specified concentrations of the compound, 1 mM 6-P-F-Asp, 100 mM HEPES (pH 8 at 22°C), 10 mM MgCl_2_, 0.1 mM EGTA, 1 mM NAD^+^, and 10 mU G6PD. Twenty-eight microliters of the assay mixture was incubated with 1 μL of the compound for 30 min at 22°C. This enzyme-inhibitor preincubation mixture was then transferred to a 384-well microplate, and the deglycase reaction was initiated by adding 1 mM 6-P-F-Asp (a substrate concentration that is nearly an order of magnitude higher than the reported *K_m_* [22]). Initial velocities were determined by monitoring the increase in the fluorescence at 450 nm using a BioTek Synergy plate reader set at 37°C (excitation at 350 nm, emission at 450 nm, and gain at 100%). The fluorescence was measured every 3 s for a total of 2 min. The rate was determined using an NADH standard curve, which was generated by plotting fluorescence versus known concentrations of NADH. For each assay, the control reaction included all components of the assay mixture except FraB, which was replaced with 1 μL buffer.
